# The implementation of grading systems for beef carcass value differentiation: the Uruguayan experience

**DOI:** 10.1093/af/vfae004

**Published:** 2024-04-16

**Authors:** Gustavo Brito, Juan M Soares de Lima, Marcia del Campo, Santiago Luzardo, Daniela Correa, Fabio Montossi

**Affiliations:** Instituto Nacional de Investigación Agropecuaria, INIA, Tacuarembó Research Station, C.P. 45000 Tacuarembó, Uruguay; Instituto Nacional de Investigación Agropecuaria, INIA, Tacuarembó Research Station, C.P. 45000 Tacuarembó, Uruguay; Instituto Nacional de Investigación Agropecuaria, INIA, Tacuarembó Research Station, C.P. 45000 Tacuarembó, Uruguay; Instituto Nacional de Investigación Agropecuaria, INIA, Tacuarembó Research Station, C.P. 45000 Tacuarembó, Uruguay; Instituto Nacional de Investigación Agropecuaria, INIA, Tacuarembó Research Station, C.P. 45000 Tacuarembó, Uruguay; Instituto Nacional de Investigación Agropecuaria, INIA, Tacuarembó Research Station, C.P. 45000 Tacuarembó, Uruguay

**Keywords:** beef, meat quality, Uruguayan carcass grading, video image analysis

**Implications**

- The Uruguayan beef industry is moving from a subjective beef carcass grading system to a video image analysis (VIA). Different studies contributed to this. Three Uruguayan Beef Quality Audits showed that 80% of the carcasses received the same muscle conformation and fatness score. This lack of discrimination associated suggested the need to develop a more discriminatory method of sorting carcasses into uniform marketing groups. The beef marketing system in Uruguay is based on hot carcass weight and visual degree of fat cover, creating a price grid in which the heaviest carcasses with fat grade 2 are rewarded, achieving the requirements of markets.
- Research has been conducted in Uruguay using ultrasound of live animal and VIA of hot and chilled carcasses to better predict red meat yield. The results from this research will be discussed.
- As the meat industry moves toward these concepts, a payment system that remunerates the individual animal merit is necessary, allowing the producer to undertake the relevant changes.

## Introduction

Carcass classification and grading system constitutes a communication mechanism between farmers, processors, and consumers, being able to determine the type of animal required by the market. It aims to define carcasses, cuts and meat quality using homogeneous criteria, grouping them into different categories, and describing the value of these in useful terms for the meat industry.

As for any other commodity, this is a real market need, which is even more accentuated in the meat industry, given by great heterogeneity among animals, carcasses, and cuts. The national beef slaughter in 2022 was of 2,400,000 animals, being 50% of the animals were steers, followed by 35% of cows, and 13% of heifers. The age at slaughter is different for each sex category. For steers, 11% were 0 teeth (th), 30%: 2 th, 29%: 4 th, 14%: 6 th, and 16%: 8 th. Forty-two percentage of heifers had 2 th, 40%: 4 th, and 18%: 0 th. Meanwhile in cows, 90% were 8 th and the remaining 10%: 6 th.

There was a predominance of British breeds that represent approximately 65% of the national slaughter, with the other 35% being dairy, cross indicus, and continental breeds. Eighty-two of the cattle came from pasture-based fattening, both improved and rangelands conditions, and the other 18% from feedlot (steers and heifers <4 teeth).

By carrying out this grading, it is possible to “improve efficiency and marketing along the entire meat chain,” promoting a better product segmentation by quality, aligning supply and demand.

These grading systems must be dynamic and evolutive, lined up to the changes of market requirements (domestic and export), processing the industry updates (e.g., automatization, traceability, certification, reducing wastage, etc.), and the production systems characteristics (breeds, animal categories, age, weight, level of fatness, technology uptake, etc.), among other factors.

## Brief History

In Uruguay, beef grading activities began in 1951. The first Official System, in force since January 1, 1956, used the letters of the word ORIENTAL to classify the different carcass qualities.

A new Official System came into force on January 1, 1976. The fundamental change in this system was the implementation of conformation and finishing attributes. The different conformations degrees were identified by the letters I, N, A, C, U, and R (I: from large to R: lack of muscle development) and for finishing five grades were stipulated (0, 1, 2, 3, and 4, representing the lack of fat up to over-finished carcasses). In 1997, a new Official System for Beef Classification and Grading was approved (resolution 65/97). The responsibility of applying the new system was left to the processor’s personnel. But, in 1999, this task was also taken officially by the staff of the Animal Industry Division belonging to the Uruguayan Ministry of Livestock, Agriculture and Fisheries (MGAP), not existing instances of cross-checking between slaughter plants. As far as classification is concerned, new grading categories were proposed based on sex and age due to their important influence on meat yield and meat sensory quality. The conformation and fat-finishing categories of carcasses are still maintained nowadays. This classification represented the first carcass quality measure applied within national regulations, which established limitations for the slaughter of some categories (Decree 401/018). International protocols require certain categories (Hilton quota: Regulation EU 593/2013 and EU 2287/2016).

The Electronic Information System of the Meat Industry (SEIIC, National Meat Institute of Uruguay [INAC]), which enhances the recording information platform of the national livestock, Decree 310/016 (2016) incorporated the Automated Typing System (SAT, Normaclass MAC 10, Scott Automation). This system is based on video image analysis (VIA) technology to automatically determine the carcass characteristics. This follows the criteria of the Official Beef Grading System, where the equipment is installed in the post-dressing slaughter line and immediately before the hot carcass scale. The aim is to provide objectivity and to standardize the carcass grading criteria associated with the degree of muscle development (extending up to 12 categories for a closer approximation to the content of saleable meat and/or valuable cuts) and carcass finishing grade (use of 10 categories).

## Research Studies on Carcass and Meat Grading in Uruguay

### Uruguayan Beef Quality Audits

Over the last 20 years, four National Beef Quality Audits have been conducted in Uruguay, being the fourth in progress. The first Uruguayan Beef Quality Audit (UBQA) was conducted in 2002, in a collaborative project among Colorado State University (CSU), INAC, and National Agricultural Research Institute (INIA). This was an important benchmark to identify and measure the quality of animals, carcasses, cuts, and meat in the Uruguayan beef industry (de Mattos et al., 2003). Most of the 2002 UBQA findings were used as training practices for producers and packers, mainly those related to animal handling practices associated with animal welfare and product quality. Meanwhile, new certified breed branded programs were developed for Hereford and Angus (Angus Grassfed Breed Verification Protocol, 2021), focusing on animals age and on meat quality attributes (marbling and tenderness levels), where the use of concentrate in the diet increased. Tracking these mentioned changes could improve the quality and consistency of the Uruguayan cattle and identifying current quality issues. The opportunity to measure quality attributes between periods and seasonal effects are contributing factors for the implementation of accurate and consistent grading systems.

In the present article, some information of the 2013 UBQA related to carcass assessments is presented. Beef carcasses were selected randomly for determination of hot carcass weight (HCW), application of the Official Grading System (INAC, 1997), USDA beef quality grade factors (overall maturity and marbling, USDA, 1997), rib eye area (REA, measured by blotting paper), and fat thickness (FTC), both measured at ribbing between 10th and 11th rib.

Tables 1 and 2 show the carcass conformation and finishing results from the application of the Official Grading System (INAC,1997) for each category under study and for the total number of carcasses evaluated. Regarding these variables, the values for conformation indicated that 86.3% of the carcasses were graded A. In terms of fat-finishing, 79.6% were graded 2. In the 2007 audit, 83.2% and 70.8% of the carcasses were graded A in conformation and 2 in fat-finishing (Brito et al., 2010; 2011), respectively.

**Table 1.** Distribution (%) of carcass conformation grades (Official Beef Grading System) from the 2013 UBQA according to the beef category and total carcasses evaluated

Grade	Steers	Cows	Heifers	Total
I	0.1	—	—	0.1
N	4.7	0.2	0.4	3.1
A	88.0	83.4	94.6	86.3
C	6.8	13.3	3.6	9.1
U	0.4	2.4	0.9	1.1
R	—	0.8	0.6	0.3
Carcasses	13,449	7,237	1,358	22,044

**Table 2.** Distribution (%) of carcass fat-finishing grades (Official Beef Grading System) from the 2013 UBQA according to the beef category and total carcasses evaluated

Grade	Steers	Cows	Heifers	Total
0	1.4	3.9	1.6	2.3
1	13.3	15.7	11.1	14.5
2	81.9	77.8	85.1	79.6
3	3.3	3.4	2.1	3.4
4	—	0.2	0.1	0.1
Carcasses	13,449	7,237	1,358	22,044

The average values of hot carcass weight obtained in the different categories did not show great differences with the values resulting from the first two audits (270.6 and 263.1 kg in steers, 221.7 and 218.6 kg in cows, and 199.2 and 203.7 kg in heifers, for 2002 and 2008, respectively; Brito et al., 2011). Higher average hot carcass weights were observed in the third audit (Brito et al., 2017). The authors observed that 50% of the steer carcasses were above 271.3 kg (Table 3). Analyzed by category, the same table shows the cross-sectional area of the longissimus dorsi muscle at the level of the 10th to 11th rib, were: overall average of REA = 59.9 cm^2^, steers = 62.9 cm^2^, cows = 55.7 cm^2^, and heifers = 57.0 cm^2^. Those values for the 2008 audit were: 60.9, 52.9, and 52.4 cm^2^ for steers, cows, and heifers, respectively. FTC overall average was 9.3 mm. By category, the averages for this variable were 9.3, 9.5, and 8.6 mm for steers, cows, and heifers, respectively. These values are similar to those reported in the two previous audits.

**Table 3.** Means and SD (in brackets) for hot carcass weight (HCW), FTC, and REA by beef category (2013 audit)

Traits	Steer	Cow	Heifer
HCW, kg)	276.1 (38.4)	224.7 (33.6)	207.2 (33.3)
FTC, mm	9.3 (5.3)	9.5 (4.9)	8.6 (5.7)
REA, cm^2^	62.9 (9.3)	55.79 (8.0)	57.0 (8.8)

Marbling is evaluated by the amount and distribution of the intramuscular fat in the ribeye muscle at the cut surface after the carcass has been ribbed between the 12th and 13th ribs and at least 10 min bloom time (USDA, 1997). In Uruguay, marbling is recorded between the 10th and 11th ribs since quartering is normally performed at this site.

Frequencies of marbling scores, carcass maturity, and USDA Quality Grade in steers are shown in Table 4. Compared to the 2008 audit, “small” marbling score (Sm) increased from 14.5% to 26.0% in the 2013 audit, meanwhile, “slight” (Sl) marbling score did not change (49.4% vs. 48.6%) between audits. A decrease in the “traces” (Tr) score was observed from the second (31.5%) to the third audit (15.1%).

**Table 4.** Frequencies (%) of skeletal maturity USDA steers classified by dentition (2013 UBQA, *n* = 2,500)

Skeletal maturity	Steers classified by dentition-teeth				
	0	2	4	6	8
A	98.2	91.5	77.7	66.1	40.9
B	1.8	8.0	21.1	31.8	52.2
C	0	0.5	0.6	1.4	5.3
D	0	0	0.6	0.7	1.6

USDA maturity levels of carcasses, A most (youthful), B, C, D, and E (oldest).

Seventy-five percent of the carcasses ranged between the Sl and Sm marbling scores and 50% of these were steers with 8 teeth. Intramuscular fat is deposited later in the animal (Di Marco, 1994), so it would be expected that the highest marbling scores would be found in older animals. However, other variables such as genetic, nutrition, and management that also explain the intramuscular fat content.

An improvement was observed in marbling scores across the three audits. Cattle fattening has improved in recent years in response, partially, to the European Union’s (EU) 481 Quota (High Quality Beef Grain-Fed beef) requirements for which producers are incorporating grain and accelerating feed conversion to facilitate the marketing of younger cattle.

Maturity refers to the physiological age of the animal rather than the chronological age measuring the degree of cartilage ossification of the split chine bones, bone characteristics, lean color, and ribeye muscle texture, although color and texture could be affected by other postmortem factors.

In the 2013 UBQA, most of steer carcasses (62.8%) were graded as A and 33.5% of them were classified as B for skeletal maturity according to the USDA QG scale. Crossing this information with dentition, 66% and 41% of 6 and 8 teeth steers, respectively, showed an A physiological maturity (Table 4). It could be inferred that beef cattle growth patterns given by the Uruguayan production systems based on pastures during the first stages of life would affect the ossification level.

Applying the USDA Quality Grade System (skeletal maturity and marbling), most of the Uruguayan steers fall into the Standard (34.7%) and Select (23.3%) grades, and only 18.7% of the steers reached Choice, including low Choice (del Campo et al., 2016; Brito et al., 2017). The 2022/2023 UBQA still in progress will yield new results in this regard and presumably, the percentages of Choice carcasses would increase due to a larger proportion of steers that are currently finished on high-concentrate diets.

According to the results obtained in the 2013 UBQA, the application of the Uruguayan Official Classification and Grading System (INAC, 1997) has shown some limitations to objectively segregate the value of carcasses in Uruguay. Automation of the national evaluation system would address the subjectivity limitations for the carcass grading, giving greater value for discrimination and transparency to the process, as well as providing a more accurate, reliable, and powerful tool. This reinforces the necessity to generate more research evidence. VIA systems, developed and tested to effectively predict beef carcass yield, are based on taking digital images of the whole side of a hot carcass or the cross-section of the rib after carcass cooling.

### Meat yield prediction using a carcass video imaging system

The objectives of this study conducted in 2002 in collaboration among CSU, Research Management Systems (RMS), INIA, and INAC were to evaluate the effectiveness of the VIAScan Beef Carcass System (HCS, hot carcass system) and the Computer Vision System (CVS; chilled carcass system) independently or in combination to predict the meat yield (percentage of valuable cuts) of representative Uruguayan beef carcasses. The information about this study was already reported by Vote et al. (2009) and it was adapted for the present article.

Descriptive statistics of carcass characteristics are presented in Table 5. Of all carcass traits, adjusted FTC showed the greatest variation (CV = 66.3%), while HCW, REA, USDA preliminary yield grade, and final yield grade (YG) showed similar variation (CV = 18.3%, 16.9%, 19.1%, and 22.6%, respectively). This helps to explain why the percentage of fat trimmings was the most variable among the calculated yields.

**Table 5.** Descriptive statistics for carcass traits (*n* = 288)

Parameters	Average	SD	Max.	Min.	C.V.
HCW, kg	224.6	41.2	149.0	374.2	18.3
REA, cm^2^	49.7	8.4	30.5	85.3	16.9
Adjusted FT, mm	8	5.1	0	25	66.3
Preliminary USDA YG	2.9	0.6	2.0	5.0	19.1
USDA YG	2.8	0.6	1.3	4.7	22.6

C.V., coefficient of variation.

The results for predicting saleable red meat yield using the INAC Official Grading System and USDA Grading System are presented in Table 6. Of all the factors considered by the Uruguayan Official System, finishing grade was the most effective in discriminating carcasses into groups differing in meat yield. Males had a higher mean in saleable meat yields than females (*P* < 0.05, data not shown). A coefficient of determination (*R*^2^ = 0.25) was obtained for predicting saleable meat yield, with the variables finishing and sex that comprised the model. Using preliminary adjusted USDA YG values were the first to be included in the model and accounted for 51% of the observed variability in saleable meat yield and REA accounted for an additional 9% of the variation (Vote et al., 2009). Steiner et al. (2003) reported that the YG assigned by USDA graders at the slaughter line accounted for 55% of the variation in yield of subprimal cuts, while the YG calculated by a USDA expert grader accounted for 71%. The variation in yield explained by YG in the present study is probably smaller because the adjusted mean FTC was much lower than that found by Steiner et al. (2003).

**Table 6.** Multiple regression equations to predict saleable meat yield using the Uruguayan official grading system

Terms in model	R^2^	RMSE
Finishing (carcass fat cover score)	0.2	0.024
Finishing, sex	0.25	0.023
Adjusted preliminary YG	0.51	0.018
Adjusted preliminary YG, REA	0.60	0.017
Adjusted preliminary YG, REA, HCW	0.61	0.017

*R* ^2^, coefficient of determination; RMSE, root mean squared error.

The best equation using CVS output explained 41%, 45%, and 55% of the observed variability in saleable red meat yield with maximum FTC of 1 cm, 0.5 cm, and no external fat, respectively. Cannell et al. (1999) found that the chilled carcass VIA system (CVS) accounted for 46%, 64%, and 68% of the variation in carcass meat yield for cuts with fat thicknesses of 2.54 cm, 0.64 cm, and no external fat, respectively.

Additionally, an interesting observation between this work and other published studies is the relationship between REA and saleable meat yield in the carcass. Simple correlation values between REA and saleable meat yield were 0.27 to 0.35. Cannell et al. (1999; 2002) found simple correlation values of 0.59 and 0.63 with the percentage of cuts with a thickness of 0.64 cm, respectively. This could be explained by Uruguayan cattle breed consistency.

The application of CVS or HCS video imaging technology individually to predict saleable meat yield did not differ from the USDA carcass classification for the population of animals analyzed in this study. The results are consistent with those reported by Cannell et al. (1999; 2002). The *R*^2^ values for predicting carcass meat yield improved from 0.64 to 0.69 in the three studies mentioned, for using both video imaging systems.

The HCW used alone accounted for most of the variation in individual cut weight and adding the CVS and HCS variables provided a small (3% to 6%) additional explanation to the observed variation in individual cut weight.

Carcass characteristics and meat instrumental tenderness (Warner-Bratzler shear force [WBSF]) values of four groups using the Official System based on sex and dentition are presented in Table 7. As expected, mature cow carcasses received the greatest (P < 0.01) skeletal maturity scores, with steers and heifers in grade A of maturity. These results are in line with those reported in UBQA. The average levels of marbling were Sl for the different categories. Striploin steaks (longissimus lumborum) aged at 2 °C until 14 d postmortem from mature cow carcasses had the greatest WBSF values (*P* < 0.01).

**Table 7.** LS means ± SD (in brackets) of USDA QG traits and WBSF values for different categories according to the Official Grading System

Traits	Young steer	Mature steer	Heifer	Mature cow
USDA marbling score	314.7b (55.1)	357.1a (81.1)	324.5ab (80.4)	338.5ab (122.3)
USDA skeletal maturity	64.5b (26.5)	89.8b (70.2)	76.8b (79.9)	326.6a (122.3)
WBSF, kg	3.6b (1.1)	3.3b (0.8)	3.4b (1.2)	4.2a (1.4)

Numerical scores for USDA marbling degrees: small (Sm) = 400 to 499, slight (Sl) = 300 to 399, traces (Tr) = 200 to 299. Numerical scores for USDA skeletal maturity degrees: A = 0 to 99, B = 100 to 199, C = 200 to 299, D = 300 to 399, E = 400 to 499. Level of significance: a < 0.01, b <0.05

The results of this study indicated that the prediction of Uruguayan beef yield with the HCS system is similar or better than values published in other countries, even though the average FTC included in other studies was higher. The predictive ability of the CVS was slightly lower than published research from other countries, which may be due to the type of cattle included in the study. The use of a dual-component VIA system could increase the accuracy of predicting saleable meat yield from Uruguayan beef carcasses.

## Conclusion

Relatively stable market conditions would make it possible to transmit “commercial signals” to define the meat characteristics of economic interest that differentiate groups of animals for different market values. The specifications in the cuts that affect value (cuts that discriminate value) are the weight of these cuts (rump and loin), fat cover degree, and pH, mainly. According to the studies presented, although weight (live weight or carcass weight) is the main variable to be considered for models to predict the main cut weights as well as for meat yield, the importance of quantifying certain meat characteristics (REA and thickness of subcutaneous fat) both in the live animal (using the ultrasound technique) and in the carcass (using the VIA system) will provide a better definition of the finishing point of the animals at slaughter and more reliable prediction equations.

The variation found in the REA and its effect on the prediction of these variables (weight and cut yield) highlights the genetic factor and its potential for expression, either by using breeders with objective information on this variable (EPD for REA) or by making strategic crosses.

Market demands for certain quality attributes and the differential price that this represents, lead to the inclusion of these methodologies to evaluate meat attributes such as marbling, meat and fat color, and palatability (juiciness, tenderness, and flavor).

## About the Authors

**Gustavo Brito** is a principal research scientist working for the National Agricultural Research Institute of Uruguay in Extensive Livestock Systems Program and Agrifoods Unit. He earned his master and PhD at The University of Georgia (United States). He was the Regional Director of INIA Tacuarembó. His work has been mainly focused on the effects of production systems and nutrition on carcass and meat quality (beef and lamb), the use of objective measurements for beef grading and predicting meat yield and quality and the nutritional attributes of Uruguayan beef. **Corresponding author**: gbrito@inia.org.uy

**Juan Manuel Soares de Lima** is a principal research scientist working for the National Agricultural Research Institute of Uruguay, in both Extensive Livestock Systems Programme and the Applied Economy Unit. He earned his PhD at Universidad Politécnica de Valencia, Spain. His expertise area is related to modeling livestock production systems to analyze the impacts of technologies, genetics, and managing practices on production, economics, and environments of these systems.

**Marcia del Campo** is an animal scientist at INIA Uruguay. His research lines are mainly focused on animal welfare at the farm level in beef cattle and lamb, as well as the impact of handling and welfare on carcass and meat quality. She earned her MSc and PhD at the Polytechnic University of Valencia, Spain.

**Daniela Correa** is an agricultural engineer and Master in Agricultural Sciences from the University of the Republic (UdelaR). She has participated in different projects related to carcass and meat quality as an assistant researcher. She is currently a pre-doctorate in animal science, studying meat aging methods from different feeding systems and their effect on physicochemical, microbiological, proteomics, and volatile compounds.

**Santiago Luzardo** is a research scientist working for the National Agricultural Research Institute of Uruguay. He earned his PhD at Colorado State University. His work has been mainly focused on the effects of production systems, management, and nutrition on carcass and meat quality in beef cattle and lamb. His most recent work has been more closely related to meat shelf life.

**Fabio Montossi** is a main research scientist working for the National Agricultural Research Institute of Uruguay. He earned his PhD at Massey University (New Zealand) and did his sabbatical studies at the Colorado State University (United States). He was the Director of the Meat and Wool National Research Program and served as the National Director at INIA. His work has been mainly focused on nutrition, plant–animal relationship and meat quality and wool in cattle and sheep and also has as areas of interest the production, transfer, and innovation systems.
